# Development of a Standardized Protocol to Measure the (An)aerobic Capacity on a Roller Ergometer Among Wheelchair Athletes

**DOI:** 10.1002/ejsc.12275

**Published:** 2025-03-16

**Authors:** Rowie J. F. Janssen, Riemer J. K. Vegter, Han Houdijk, Lucas H.V. van der Woude, Sonja de Groot

**Affiliations:** ^1^ University of Groningen University Medical Center Groningen Center for Human Movement Sciences Groningen The Netherlands; ^2^ Peter Harrison Centre for Disability Sports School of Sport Exercise and Health Sciences Loughborough University Loughborough UK; ^3^ Center for Rehabilitation University Medical Center Groningen Groningen The Netherlands; ^4^ Department of Human Movement Sciences Faculty of Behavioural and Movement Sciences VU University Amsterdam The Netherlands; ^5^ Amsterdam Rehabilitation Research Center Reade Amsterdam The Netherlands

**Keywords:** aerobic exercise, anaerobic exercise, ergometry, exercise test, para‐athletes, wheelchairs

## Abstract

To evaluate and update about previous regression equations to derive standardized and individualized resistance settings for wheelchair‐specific anaerobic and aerobic capacity tests in wheelchair athletes. An isometric strength test, a sprint test, a Wingate anaerobic test (WAnT), and an aerobic graded exercise test (GXT) were performed by 43 wheelchair athletes on a computerized roller ergometer. Using previously developed regression equations, measured strength predicted anaerobic power and determined the individual's WAnT resistance. Subsequently, measured anaerobic power predicted aerobic power and determined the individual's GXT resistances. The WAnT was considered valid when peak rim velocity stayed below 3 m·s^−1^ and the GXT when the test duration was between 8 and 12 min. After testing, individual test results were used to construct new regression equations to improve predictions for anaerobic and aerobic power. The strength test turned out to be nonstatic for the five strongest athletes. Consequently, their WAnT resistance was underestimated, resulting in the highest peak rim velocities. The GXT had durations below 8 min for seven athletes. The sprint test was feasible for every athlete and showed a better prediction for anaerobic power (*R* ^2^ = 0.84). The updated regression equation to predict aerobic power from anaerobic power resulted in an *R* ^2^ of 0.78. For future testing in wheelchair athletes, it is advised to use the newly developed athlete‐specific regression equations to predict (an) aerobic power and to set adequate WAnT and GXT resistances. These standardized and individualized settings will lead to interathlete and intra‐athlete comparable measures, used for athlete monitoring or to set training guidelines.

**Summary**

- The current study revised test protocols to measure the wheelchair‐specific anaerobic and aerobic capacity in a standardized manner and tailored to individual characteristics. The revised test protocols provide athletes and coaches with interathlete and intra‐athlete comparable measurements of their anaerobic and aerobic capacity.
- The newly developed athlete‐specific regression equations accurately predict anaerobic power (*R* ^2^ = 0.84) and aerobic power (*R* ^2^ = 0.78) and should be used in the future to set appropriate resistances for Wingate and graded exercise tests.
- These outcome measures support performance monitoring, training optimization, and wheelchair configuration adjustments, all aimed at understanding and —ultimately—improve wheelchair sport performance.

## 1 Introduction

In the current format of the Paralympic games, four sports critically depend on handrim wheelchair performance: basketball, rugby, tennis, and racing (including triathlon). Although there is a large heterogeneity in impairments among athletes and wheelchairs used, all wheelchair athletes have in common that their anaerobic and aerobic capacities are important, albeit in varying ratios (Paulson et al. 2017). However, wheelchair athletes currently lack standardized wheelchair‐specific tools for objective monitoring of their anaerobic and aerobic capacities (R. J. F. Janssen et al. 2021). Today, these capacities can be measured with a computerized roller ergometer that objectively measures the power output (W) and that allows testing athletes in their own individual sports wheelchair (R. J. F. Janssen et al. 2021) (de Klerk et al. 2020a).

Since wheelchair athletes are highly heterogeneous (e.g., age, sex, disability, and sports discipline), it is difficult to determine beforehand the adequate individual resistance settings that will lead to valid anaerobic Wingate tests (WAnT) and aerobic graded exercise tests (GXT) (Veeger, van der Woude, and Rozendal 1991) (Buchfuhrer et al. 1983). This has led to varying resistance settings for the WAnT and GXT, often based on the expertise of the test leader and/or on a priori knowledge of the athletes, which hampers reproducibility, generalizability, and thus comparability among studies (R. J. F. Janssen et al. 2021). Recently, in a literature review (R. J. F. Janssen et al. 2021), we proposed a standardized, yet individualized way to determine resistance settings for the WAnT and GXT by using a test battery consisting of a wheelchair‐specific isometric strength test, sprint test, WAnT, and GXT. The first two tests, isometric strength test and sprint test, have standardized resistance settings, whereas the subsequent two tests, the WAnT and GXT, have individual resistance settings that are derived from earlier developed regression equations (T. W. J. Janssen et al. 1993). This test battery has led to feasible WAnT and GXT resistance settings in a rehabilitation population (Dallmeijer et al. 1999a), (Dallmeijer et al. 1999b) and able‐bodied participants (R. J. F. Janssen et al. 2022) (van der Woude et al. 1999). With the current study, we aim to evaluate this standardized, yet individualized test protocol for (an) aerobic capacity testing among a heterogeneous group of wheelchair athletes.

The WAnT assesses the anaerobic capacity and is a 30 s sprint test against a predetermined high resistance (Bar‐Or 1987). The proper resistance should be set individually because too low or too high velocities will limit power production as muscles have to act at too high or too low force levels (Veeger, van der Woude, and Rozendal 1991) (Hintzy et al. 2003). Using previously developed regression equations (T. W. J. Janssen et al. 1993), measured isometric strength (N) was used to predict anaerobic power output (W) and subsequently the individual's WAnT resistance was determined (Dallmeijer et al. 1999a, 1999b; R. J. F. Janssen et al. 2022; van der Woude et al. 1999). However, previous research that used isometric strength as predictor showed methodological limitations for the strongest participant (R. J. F. Janssen et al. 2022). Therefore, a standardized sprint test was added to the test battery as potential substitute for the isometric strength test in predicting anaerobic power and to set the individual's WAnT resistance.

The GXT assesses the aerobic capacity in a 1 min stepwise test protocol where the test duration is determined by start resistance and completed steps. An ideal test duration is between 8 and 12 min, that is, shorter protocols with too high resistances tend to induce muscle fatigue whereas longer protocols with too low resistances will lead to a higher body temperature, dehydration, discomfort, or ventilatory muscle fatigue (Buchfuhrer et al. 1983). Again using the previously developed regression equations (T. W. J. Janssen et al. 1993), measured anaerobic power can be used to predict aerobic power output (W), and subsequently, the individual's GXT start resistance and resistance steps were determined.

The suggested standardized test protocol potentially should allow every athlete to perform a WAnT and GXT under the proper individualized conditions (R. J. F. Janssen et al. 2021). Using regression equations, the goal is to have outcomes fit within a broad acceptable range, appreciating athletic differences in anaerobic:aerobic ratios, but staying within acceptable test boundaries. It remains unclear whether the previously developed regression equations (T. W. J. Janssen et al. 1993), based on data from male wheelchair users with a variety of spinal cord injuries, are valid for a more heterogeneous population of wheelchair athletes or whether these equations require refinement for future testing. These equations could be improved by incorporating earlier test results, such as the isometric force and sprint test or by including personal characteristics. Given the heterogeneity among wheelchair athletes, factors, such as age, sex, and sport discipline, should be considered in the regression equation to improve the accuracy of anaerobic and aerobic power predictions.

Therefore, the aim of this study was to evaluate and update previous regression equations to derive standardized and individualized resistance settings for wheelchair‐specific anaerobic and aerobic capacity tests on a computerized roller ergometer in wheelchair athletes. Resistance settings are derived from individually predicted anaerobic and aerobic power, and it is expected that with these settings, athletes attain outcomes close to their predicted values, which consequently leads to valid WAnT and GXTs. The WAnT is considered valid when peak rim velocity stays below 3 m·s^−1^. The GXT is considered valid when the test duration is between 8 and 12 min (Veeger, van der Woude, and Rozendal 1991) (Buchfuhrer et al. 1983). After testing, individual test results and personal characteristics will be used to construct new regression equations for this specific population to further improve testing of anaerobic and aerobic capacity.

## 2 Material and Methods

### 2.1 Participants

In this cross‐sectional study, Paralympic wheelchair athletes were included on a voluntary basis between March 2021 and July 2023. Athletes participated in wheelchair basketball, tennis, rugby, or racing (including triathlon) at an international level. Participant demographics can be found in Table 1. All athletes declared the absence of medical contraindications according to the Physical Activity Readiness Questionnaire (PAR‐Q) (Adams 1999). All athletes gave their written informed consent prior to testing. The local ethics committee of the Centre for Human Movement Sciences, University Medical Centre Groningen, University of Groningen, the Netherlands approved the study protocol (202000455).

**TABLE 1:** Mean and standard deviation for participant characteristics and for the outcomes of the four tests: isometric strength, sprint, WAnT, and GXT. Reported for all athletes together and separately per sport discipline.

	All athletes (28M/15F)	Rugby (11M/0F)	Basketball (3M/6F)	Tennis (6M/6F)	Racing (8M/3F)
Participant characteristics					
Age (yrs)	26 ± 7	26 ± 5	26 ± 6	26 ± 8	27 ± 10
Body mass (kg)	67 ± 12	74 ± 9	67 ± 14	64 ± 13	63 ± 9
Disability	13 SCI, 9 SB, 5 AMP, 7 CP, 9 LA	4 SCI, 5 CP, 2 LA	4 SCI, 2 SB, 1 AMP, 2 LA	1 SCI, 3 SB, 3 AMP, 5 LA	4 SCI, 4 SB, 1 AMP, 2 CP
Time since injury (yrs)	21 ± 9	20 ± 10	18 ± 8	22 ± 11	23 ± 7
Sport experience (yrs)	8 ± 6	6 ± 4	8 ± 4	13 ± 8	5 ± 5
Training/week (hrs)	18 ± 6	18 ± 5	19 ± 4	21 ± 6	13 ± 5
Isometric strength test					
F_iso_ (N)	262 ± 69	267 ± 69	250 ± 53	275 ± 59	253 ± 93
Sprint test					
PO_mean_ (W)	124 ± 66	98 ± 17	93 ± 29	97 ± 28	203 ± 84
PO_max_ (W)	839 ± 398	731 ± 322	694 ± 367	754 ± 204	1151 ± 500
*v*_mean_ (m·s^−1^)	3.6 ± 0.8	3.0 ± 0.4	3.2 ± 0.4	3.4 ± 0.4	4.6 ± 0.9
*v*_max_ (m·s^−1^)	4.7 ± 1.4	3.8 ± 0.4	4.0 ± 0.5	4.4 ± 0.6	6.6 ± 1.3
Wingate test					
P30_pred_ (W)	122 ± 34	123 ± 36	115 ± 26	129 ± 29	118 ± 46
P30_meas_ (W)	139 ± 48	125 ± 36	119 ± 35	145 ± 41	168 ± 65
Difference_pred‐meas_ (%)	−11 ± 19*	−1 ± 14	0 ± 18	−9 ± 14*	−34 ± 13*
P5 (W)	185 ± 61	170 ± 45	166 ± 51	182 ± 50	222 ± 85
v_max_ (m·s^−1^)	2.6 ± 0.4	2.4 ± 0.2	2.4 ± 0.4	2.6 ± 0.3	2.8 ± 0.5
Graded exercise test					
PO_peak‐pred_ (W)	102 ± 33	92 ± 24	87 ± 25	105 ± 28	121 ± 46
PO_peak‐meas_ (W)	90 ± 26	73 ± 17	91 ± 15	102 ± 26	94 ± 34
Difference_pred‐meas_ (%)	14 ± 23*	26 ± 18*	−6 ± 11	3 ± 16	33 ± 27*
Test duration (min)	9.8 ± 1.9	8.9 ± 1.4	11.5 ± 1.3	10.6 ± 1.6	8.3 ± 1.9
$\dot{V}$O_2peak_ (ml·min^−1^·kg^−1^)	39.3 ± 9.8	37.8 ± 14.5	33.6 ± 5.5	42.2 ± 7.5	42.6 ± 7.0

### 2.2 Measurement Setup

**FIGURE 1:**
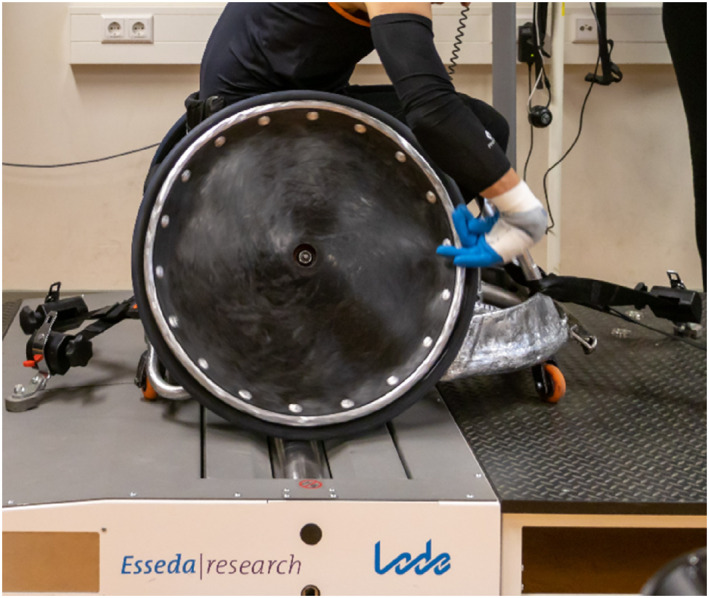
The Esseda wheelchair roller ergometer, accommodated with an athlete's sports wheelchair.

Tests were conducted by a single test leader (RJFJ) at four different locations (University Medical Centre Groningen, Reade center for Rehabilitation and Rheumatology in Amsterdam, National Sport Centre Papendal and at the University of Applied Sciences in The Hague), using an identical Esseda wheelchair roller ergometer (Figure 1, Lode BV, Groningen, The Netherlands) (de Klerk et al. 2020b). This commercial wheelchair dual roller ergometer allows for accurate measurements of tangential torque and velocity at a sampling frequency of 100 Hz, for both left‐ and right‐hand propulsion (de Klerk et al. 2020b). Before each session, the ergometer was calibrated to account for static and dynamic friction of each individual wheelchair‐athlete combination (de Klerk et al. 2020b). All athletes used their own sports wheelchair and their sport‐specific attributes (e.g., gloves and strapping, but no tennis racket). Before testing, tires of the wheelchairs were inflated up to their recommended pressure.

Heart rate (HR) and breath‐by‐breath oxygen consumption were measured using the Cosmed K5 (COSMED, Rome, Italy) or the Metamax 3B (Cortex Biophysics, Leipzig, Germany) depending on the test location. At the start of every test day, this equipment was calibrated for sensitivity and volume according to the guidelines of the used metabolic measurement system.

### 2.3 Data Analysis

All analyses were conducted with a custom‐written Python script (Worklab: a wheelchair biomechanics mini‐package (de Klerk et al. 2023)). Torque at the wheel and velocity data were directly measured from the wheelchair ergometer and filtered using a fourth order low‐pass Butterworth filter with a cutoff frequency of 10 Hz (Cooper et al. 1998). Effective isometric push force (F) at each rim side was calculated with the measured torque (M) and individual rim radius (r_r_):

(1) $$F\;\left(N\right)=M\;\left(N\cdot m\right)\;x\;r_{r}^{-1}\;\left(m\right)$$

The power output (PO) at each side was calculated from the measured torque (M), wheel radius (r_w_), and wheel velocity (v_w_):

(2) $$\text{PO}\;\left(W\right)=M\;\left(N\cdot m\right)\;*\;r_{w}^{-1}\;\left(m\right)\;*\;v_{w}\;\left(m\cdot s^{-1}\right)$$

Breath‐by‐breath analyses from the metabolic measurement systems provided data on oxygen uptake ($\dot{V}$O_2_), respiratory exchange ratio (RER), and heart rate (HR) and did not require further preprocessing. Notably, part of the data analyses was performed during testing, as the isometric strength test results used to determine the WAnT protocol settings and the WAnT outcomes informed the GXT settings. Specific calculations for the outcome measures are explained in the following section (2.4 test protocols).

### 2.4 Test Protocols

**FIGURE 2:**
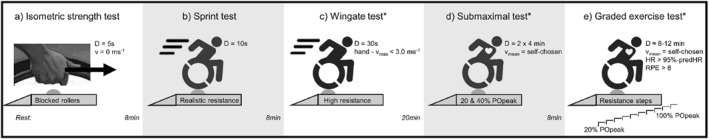
A schematic overview of the experimental protocol. Setup for the (a) isometric strength test, (b) sprint test, (c) Wingate test, (d) submaximal test, and (e) graded exercise test. D = duration, v = velocity, HR = heart rate, and RPE = rate of perceived exertion. *Resistance is individually determined.

A previously developed test battery was used in the current study (R. J. F. Janssen et al. 2022). Athletes performed five different types of tests on the wheelchair ergometer in a fixed sequence with standardized rest periods between each of the subsequent tests (Figure 2). First, an isometric strength test (3 × 5 s) was performed, followed by a 10 s sprint test, 30 s WAnT, a submaximal test (2 × 4 min), and lastly, a GXT based on a 1 min stepwise test protocol (8–12 min). Verbal encouragement was provided throughout each test. Before starting with the test battery, a warm‐up of 5 min at a self‐chosen comfortable velocity to familiarize with wheelchair propulsion on an ergometer was performed. The submaximal test was performed to gain insight in gross mechanical efficiency (van der Woude et al. 2001) and propulsion technique, yet these outcomes are beyond the scope of this study.

#### 2.4.1 Isometric Strength Test

**FIGURE 3:**
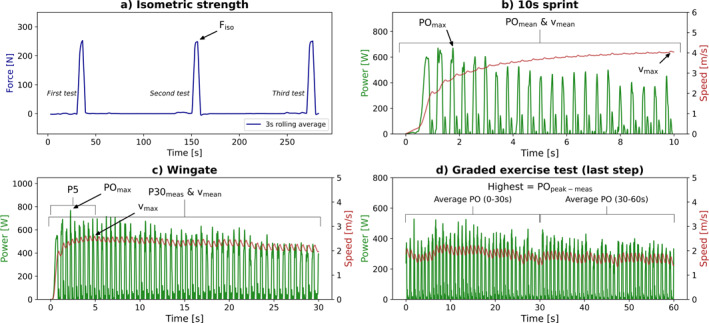
Extracted variables for the (a) isometric strength test, (b) sprint test, (c) Wingate test, and (d) the last step of the graded exercise test. Force (N) is displayed in blue, power output (W) in green, and velocity (m·s^−1^) in red.

Athletes exerted maximal force with both hands for 5 s along the tangential direction of the hand rim, repeated three times, with 2 min rest in between (Figure 2a). Court athletes (basketball, tennis, and rugby) were asked to push at the top dead center of the hand rim. Racing athletes had to push at 90^o^ from top dead center, at the front of the wheel, such as their start position during a race. Maximal isometric strength (F_iso_) was defined as the highest mean force ((N); averaged over left and right) and derived from the data with a 3 s rolling average (Figure 3a) (T. W. J. Janssen et al. 1993).

#### 2.4.2 Sprint Test

Athletes performed a 10 s sprint, from stand still, at a resistance similar to a gym court (rolling resistance coefficient (μ) = 0.012) (de Klerk et al. 2020b), repeated 2 times, with 2 min rest in between (Figure 2b). In order to be able to compare among all sports, no sport‐specific adjustments were done regarding the rolling resistance (i.e., a wooden rugby floor or an athletics track have a lower rolling resistance). Mean and maximal power output (W) (PO_mean_ and PO_max_) and velocity (m·s^−1^) (v_mean_ and v_max_) were defined as the mean power and velocity over 10 s and as the one sample highest peak, respectively (Figure 3b). Power was the sum of both arms, velocity the mean. The sprint with the highest v_max_ was chosen for further analyses.

#### 2.4.3 Wingate Test (Protocol Set up)

Athletes performed a 30 s sprint test, from stand still, at a high individualized resistance (Figure 2c). Individually predicted anaerobic power over 30 s (P30_pred_) was calculated from F_iso_ (relative to body mass) according to the regression equation below (Equation (3), (T. W. J. Janssen et al. 1993)) and multiplied with the mass of the user for the absolute P30_pred_ (Equation (4)).

(3) $$P30_{\text{pred}}\;\left(W\cdot {\text{kg}}^{-1}\right)=0.51\;*\;F_{\text{iso}}\;\left(N\cdot {\text{kg}}^{-1}\right)-0.18\;\left(R^{2}=0.75\right)$$

(4) $$P30_{\text{pred}}\;\left(W\right)=P30_{\text{pred}}\;\left(W\cdot {\text{kg}}^{-1}\right)\;*\;m_{\text{user}}\;\left(\text{kg}\right)$$

To prevent coordination problems of the upper body that may limit anaerobic power, maximal hand velocity should stay below 3 m·s^−1^ (Veeger, van der Woude, and Rozendal 1991). Therefore, a resistance coefficient was calculated that would result in the predicted P30_pred_ at a mean rim velocity of 2 m·s^−1^ (Equation (5)). Subsequently, the required resistance coefficient (μ) is determined relative to the total mass of the participant and the wheelchair (m_total_) (Equation (6)):

(5) $$v_{\text{mean}-\text{wheel}}\;\left(m\cdot s^{-1}\right)=\left(v_{\text{mean}-\text{rim}}\;\left(m\cdot s^{-1}\right)\;*\;r_{w}\;\left(m\right)\right)\;*\;r_{r}^{-1}\;\left(m\right)$$

(6) $$\text{Resistance}\;\text{coefficient}\;\left(\mu \right)=P30_{\text{pred}}\;\left(W\right)\;*\;{v_{\text{mean}-\text{wheel}}}^{-1}\;\left(m\cdot s^{-1}\right)\;*\;{m_{\text{total}}}^{-1}\;\left(N\right)$$

#### 2.4.4 Wingate Test (Outcomes)

The P30 measured during the test (P30_meas_) was calculated as the mean power over 30 s, P5 as the highest mean power over 5 s (using a rolling average technique), and POmax as the highest single sample peak (Figure 3c). Maximal rim velocity (v_max‐rim_) was defined as the highest single sample peak rim velocity. Power was the sum of the both arms, velocity the mean. The WAnT was considered valid when v_max‐rim_ < 3.0 m·s^−1^.

#### 2.4.5 Graded Exercise Test (Protocol Setup)

Finally, athletes performed a GXT to exhaustion (Figure 2e). The resistance increased every minute while maintaining a constant self‐chosen comfortable velocity. A computer screen in front of the athletes provided visual feedback on the actual and target velocity (de Klerk et al. 2020b). Predicted aerobic power (PO_peak‐pred_) was calculated from the P30_meas_ (relative to body mass) according to the following regression equation (T. W. J. Janssen et al. 1993):

(7) $${\text{PO}}_{\text{peak}-\text{pred}}\;\left(W\cdot {\text{kg}}^{-1}\right)=0.67\;*\;P30_{\text{meas}}\;\left(W\cdot {\text{kg}}^{-1}\right)+0.11\;\left(R^{2}=0.81\right)$$

By using Equations (4) and (6) and replacing P30 for PO_peak_, the initial resistance was set equal to 20% of the PO_peak‐pred_. In every subsequent step, the resistance increased by 10% of the difference between the starting load and PO_peak‐pred_, so that exhaustion and the actual PO_peak_ would be reached around minute 10. During the test, HR and $\dot{V}$O_2_ were measured continuously (see description at 2.2 measurement setup). The test was terminated when the participant could no longer maintain the required velocity (0.28 m·s^−1^ below target velocity).

#### 2.4.6 Graded Exercise Test (Outcomes)

PO_peak‐meas_ was calculated as the highest mean power (sum of both arms) over 30 s in the final 1 min block (Figure 3d) (Kouwijzer et al. 2019). Peak oxygen uptake ($\dot{V}$O_2peak_) and peak respiratory exchange ratio (RER_peak_) were defined as the highest 30 s values. HR_peak_ was the one sample highest peak. At the end of the test, peripheral and central rate of perceived exertion (RPE) were asked using a numeric rating scale from 1 to 10 (Pandolf et al. 1984). The mean of the peripheral and central RPE form the overall RPE (Goosey‐Tolfrey et al. 2014). The GXT was considered valid when the test duration was between 8 and 12 min. Secondary criteria were to reach two of the following three criteria: a RER_peak_ ≥ 1.10, a HR_peak_ ≥ 95% predicted HR_peak_ (200–age), and a RPE_peak_ ≥ 8 (Goosey‐tolfrey 2008). Athletes with a lesion level above T5 may have a loss of sympathetic outflow to the heart and their maximal heart rate is thus reduced; therefore, the HR criterion was neglected in these athletes (Paulson et al. 2017).

### 2.5 Statistical Analysis

The mean and standard deviation for every outcome of the four tests was calculated for all athletes together and separately per sport discipline. To check for systematic differences between the predicted and measured P30 and PO_peak_, a paired *t*‐test or Wilcoxon signed rank test (depending on the Shapiro–Wilk test for normality) was performed for all athletes together and separately per sport discipline; significance was set at *p* < 0.05. To obtain more insight in the potential deviation among the individually predicted and measured values, scatter plots were made with 20% deviation boundaries relative to the line of identity. These boundaries were based on a test duration of 10 ± 2 min (=20%) to reach PO_peak_ in the GXT and P30 follows these boundaries (Buchfuhrer et al. 1983).

Multiple linear regression analysis was applied to predict anaerobic power (P30_meas_) and aerobic power (PO_peak‐meas_). To minimize the influence of body mass as an underlying reason for the associations studied, all force and power output variables in the regression were expressed per kilogram body mass. Participant characteristics and earlier test results from that day were used as candidate independent variables: sex (male = 1 and female = 0), age (yrs), practiced sport (rugby, basketball, tennis, and racing, encoded as three dummy variables with tennis as reference sport), time since injury (yrs), experience in sport (yrs), training hours per week (hrs), isometric strength (F_iso_), and sprint results (v_mean_, v_max_, PO_mean_, or PO_max_) and in case of the aerobic power prediction: also the WAnT results (P30_meas_, P5 and PO_max_). Only independent variables that showed a significant univariate association (*p* < 0.10) with respectively P30_meas_ and PO_peak‐meas_ were added to the model, after which a backward regression was performed until only significant determinants remained (*p* < 0.05). Due to the high correlation (> 0.8) among the sprint variables (v_mean_, v_max_, PO_mean_, or PO_max_) and among the WAnT variables (P30_meas_, P5, and PO_max_), they were not added to the same model and only the variable with the highest association (based on *R* ^2^) with P30_meas_ or PO_peak‐meas_ was entered in the regression. Python 3.8 (Python Software Foundation) was used for all analyses.

## 3 Results

This study included 43 wheelchair athletes: 11 rugby athletes, 9 basketball athletes, 12 tennis athletes, and 11 racing athletes. Table 1 shows participant characteristics and outcomes of the isometric strength test, sprint test, WAnT, and GXT for all athletes together and separately per sport discipline.

### 3.1 Isometric Strength Test

**FIGURE 4:**
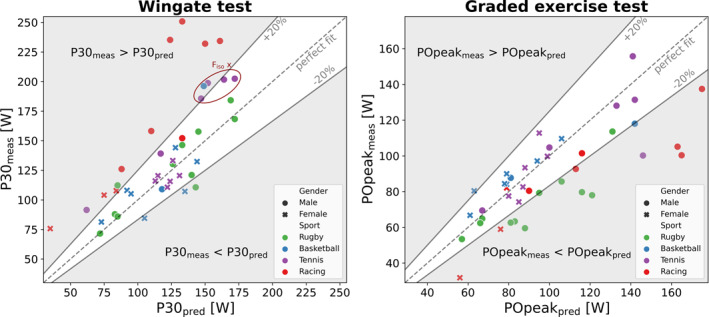
Comparison of the individual predicted P30 (left graph) and PO_peak_ (right graph) with the actual measured P30 and PO_peak_. Dotted lines correspond to a perfect fit and the solid lines represent 20% boundaries. Athletes, who were too strong for the isometric strength test, are circled in the left graph (F_iso_ x).

The isometric strength test was feasible in 38 individuals, but five athletes surpassed the ergometer force limit (four tennis athletes and one basketball athlete; Figure 4). The electrical brakes of the rollers of the wheelchair ergometer were not strong enough, resulting in unintended motion of the wheels and rollers. Although the other athletes could perform the test according to requirements, it showed some additional difficulties. Racing athletes in the current study used solid gloves (Rice et al. 2016) with which they are hitting the wheel during a push, but which prevented them from firmly gripping their handrims. To prevent their hands slipping of the rims during the strength test, they had to produce extra force in the medial direction, not measured using the ergometer. Furthermore, three rugby athletes (with the highest spinal cord injury level) could not push at the top dead center of the wheel but pushed at −90° from top dead center (at the back of the wheel), similar to their starting position. Lastly, some athletes had sweaty hands or smooth wheel surfaces, which also led to slightly moving hands during the isometric strength tests. Despite these difficulties, all forces were still used to predict anaerobic power and determined the individual's WAnT resistance. For the whole group, isometric strength ranged from 84 to 421 N, both values achieved by racing athletes.

### 3.2 Sprint Test

The sprint test could be performed by all athletes. The transfer of the data from the ergometer to the computer failed once, which resulted in missing sprint data for one tennis athlete. PO_mean_ ranged from 57 W for a basketball athlete to 368 W for a racing athlete. PO_max_ ranged from 279 W for a rugby athlete to 2207 W for a racing athlete. v_mean_ ranged from 2.3 m·s^−1^ for a rugby athlete to 6.2 m·s^−1^ for a racing athlete. v_max_ ranged from 3.1 m·s^−1^ for a rugby athlete to 9.0 m·s^−1^ for a racing athlete.

### 3.3 Wingate Test

The WAnT could be performed by all athletes. Individual anaerobic power (P30) is presented in Figure 4 and ranged from 72 W for a rugby athlete to 251 W for a racing athlete. P5 ranged from 97 W for a rugby athlete to 337 W for a racing athlete. The Wilcoxon signed rank test showed that the P30_meas_ was significantly higher (11%) than the P30_pred_ for the whole group (*p* < 0.01). More specifically, the P30_meas_ was significantly higher (9%) than the P30_pred_ in tennis athletes (*p* = 0.023) and 34% higher in racing athletes (*p* = 0.001). Basketball and rugby athletes showed no significant difference between the P30_meas_ and the P30_pred_. On an individual level, P30_meas_ was higher than predicted in three tennis athletes (> 20%), one basketball athlete, one rugby athlete, and in nine racing athletes (Figure 4). From these athletes, three racing athletes had a maximal rim velocity above the set limit of 3.0 m·s^−1^ and, therefore, test results were considered invalid. P30_meas_ was lower than predicted (< 20%) in one rugby athlete and two basketball athletes (Figure 4).

### 3.4 Graded Exercise Test

The GXT could be performed by all athletes. A technical error occurred once, which resulted in missing data for one racing athlete. During the GXT, court athletes had a velocity between 1.4 and 2.5 m·s^−1^ and racing athletes between 4.2 and 6.9 m·s^−1^. Individual aerobic power (PO_peak_) is presented in Figure 4 and ranged from 32 W for a racing athlete to 156 W for a tennis athlete. $\dot{V}$O_2peak_ ranged from 17.9 mL·min^−1^·kg^−1^ for a rugby athlete to 54.0 mL·min^−1^·kg^−1^ for a tennis athlete. The paired *t*‐test showed that the PO_peak‐meas_ was significantly lower (14%) than the PO_peak‐pred_ for the whole group (*p* < 0.01). More specifically, the PO_peak‐meas_ was significantly lower (26%) than the PO_peak‐pred_ in rugby athletes (*p* < 0.01) and 33% lower in racing athletes (*p* = 0.01). Basketball and tennis athletes showed no significant difference between PO_peak‐meas_ and PO_peak‐pred_. On an individual level, PO_peak‐meas_ was lower than predicted (<20%) in six rugby athletes, one tennis athlete, and six racing athletes (Figure 4). From these athletes, seven athletes had a test duration of 7 min, which was below the set limit of 8 min and, therefore, these test results were considered invalid. Seven athletes had an RER_peak_ below 1.10 and three athletes had a mean RPE below 8. All athletes from whom the HR was considered reached the HR_peak_ criteria (≥ 200–age). All athletes met two out of these three criteria for maximal aerobic exercise.

### 3.5 Multiple Regression Results

#### 3.5.1 Regression to Predict Anaerobic Power (P30)

**FIGURE 5:**
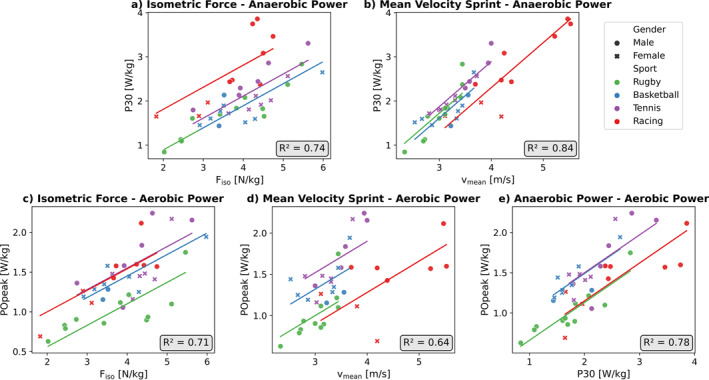
Visualization of the multiple linear regression analyses to predict the anaerobic and aerobic power for every sport (green = rugby, blue = basketball, purple = tennis, and red = racing). (a, b) Anaerobic power is predicted using respectively isometric strength and mean sprint velocity. (c–e) Aerobic power is predicted using respectively isometric strength, mean sprint velocity, and anaerobic power.

Anaerobic power (i.e., P30_meas_) showed, in descending order, a significant univariate association with sprint‐v_mean_ (*R* ^2^ = 0.77 and *p* < 0.01), F_iso_·kg^−1^ (*R* ^2^ = 0.45 and *p* < 0.01), practiced sport (*R* ^2^ = 0.31 with racing versus tennis (*R* ^2^ = 0.21 and *p* < 0.01) and rugby versus tennis (*R* ^2^ = 0.12 and *p* < 0.05) as significant determinants), and sex (*R* ^2^ = 0.08 and *p* < 0.1). Two multiple regression analyses were performed, one based on the outcome of the isometric strength test (F_iso_·kg^−1^) and the other on the result of the sprint test (v_mean_). Practiced sport remained as independent variable in both models and this resulted in an *R* ^2^ of 0.74 and 0.84, respectively (Table 2, Figure 5a,b).

**TABLE 2:** Results from the multiple regression analysis to predict anaerobic power (i.e., P30) and aerobic power (i.e., POpeak).

Regression equation		*p*‐value coefficients	Cumulative *R* ^2^	*n*
Predict P30 by isometric strength test (Figure 5a)				
P30 (W·kg^−1^) =	0.11	0.70		42
	+0.50 × F_iso_ (N·kg^−1^)	< 0.01		
	+0.70 × racing (1 = yes, 0 = no)	< 0.01		
	–0.22 × basketball (1 = yes, 0 = no)	0.19		
	–0.22 × rugby (1 = yes, 0 = no)	0.17	0.74	
Predict P30 by sprint test (Figure 5b)				
P30 (W·kg^−1^) =	–1.19	< 0.01		41
	+1.02 × sprint‐*v*_mean_ (m·s^−1^)	< 0.01		
	–0.59 × racing (1 = yes, 0 = no)	< 0.01		
	–0.26 × basketball (1 = yes, 0 = no)	0.06		
	–0.15 × rugby (1 = yes, 0 = no)	0.24	0.84	
Predict PO_peak_ by isometric strength test (Figure 5c)				
PO_peak_ (W·kg^−1^) =	0.47	< 0.05		42
	+0.27 × F_iso_ (N·kg^−1^)	< 0.01		
	–0.01 × racing (1 = yes, 0 = no)	0.95		
	–0.1 × basketball (1 = yes, 0 = no)	0.35		
	–0.45 × rugby (1 = yes, 0 = no)	< 0.01	0.71	
Predict PO_peak_ by sprint test (Figure 5d)				
PO_peak_ (W·kg^−1^) =	0.38	0.15		41
	+0.38 × sprint‐*v*_mean_ (m·s^−1^)	< 0.01		
	–0.62 × racing (1 = yes, 0 = no)	< 0.01		
	–0.2 × basketball (1 = yes, 0 = no)	0.1		
	–0.52 × rugby (1 = yes, 0 = no)	< 0.01	0.64	
Predict PO_peak_ by Wingate test (Figure 5e)				
PO_peak_ (W·kg^−1^) =	0.56	< 0.01		41
	+0.47 × P30 (W·kg^−1^)	< 0.01		
	–0.35 × racing (1 = yes, 0 = no)	< 0.01		
	–0.01 × basketball (1 = yes, 0 = no)	0.90		
	–0.37 × rugby (1 = yes, 0 = no)	< 0.01	0.78	

Abbreviations: *F*_iso_ = isometric strength, *n* = number of athletes, P30 = anaerobic power, PO_peak_ = aerobic power, *R* ^2^ = explained variance, and sprint‐v_mean_ = mean velocity sprint test.

#### 3.5.2 Regression to Predict Aerobic Power (PO_peak_)

Aerobic power (i.e., PO_peak‐meas_) showed, in descending order, a significant univariate association with WAnT‐P30·kg^−1^ (*R* ^2^ = 0.58 and *p* < 0.01), F_iso_·kg^−1^ (*R* ^2^ = 0.50 and *p* < 0.01), practiced sport (*R* ^2^ = 0.34 with rugby vs. tennis (*R* ^2^ = 0.30 and *p* < 0.01) as significant determinant), and sprint‐v_mean_ (*R* ^2^ = 0.31 and *p* < 0.01). Three multiple regression analyses were performed, one based on the outcome of the isometric strength test (F_iso_·kg^−1^), one based on the result of the sprint test (v_mean_), and the last one based on the result of the WAnT (P30·kg^−1^). Practiced sport remained as independent variable in all three models and this resulted in an *R* ^2^ of 0.71, 0.64, and 0.78, respectively (Table 2, Figure 5c–e).

## 4 Discussion

The current study evaluated previous regression equations to derive standardized and individualized resistance settings for wheelchair‐specific anaerobic and aerobic capacity testing on a computerized roller ergometer in 43 wheelchair athletes from four different sports with a large range of impairments and wheelchairs. By using earlier developed regression equations, anaerobic (i.e., P30) and aerobic (i.e., PO_peak_) power and resistance settings for the WAnT and GXT were accurately predicted for the majority but not for all athletes. In order to achieve more proper individualized resistance settings, the current study updated the previous regression equations toward the current study sample, that is, a heterogeneous group of elite wheelchair athletes. Because of methodological limitations of the isometric strength test, the current study created a new regression equation based on the sprint test, which is advised to use in future testing as predictor for anaerobic power and to set the individual's WAnT resistance. GXT resistance settings led to too low test durations for some rugby and racing athletes. The current study updated the previous regression equations (T. W. J. Janssen et al. 1993) to a new sport‐specific regression equation, which is advised to use in future testing as predictor for the aerobic power and to set the individual's GXT resistance in the population of wheelchair athletes.

### 4.1 Wingate Anaerobic Test

Anaerobic power was accurately predicted from isometric strength in basketball and rugby athletes and led to valid WAnT resistances. In tennis and racing athletes, measured anaerobic power was higher than predicted. This was caused by too low WAnT resistances resulting into the highest peak rim velocities during the WAnT (between 2.8 and 3.6 m·s^−1^). This can be explained by the methodological difficulties of the isometric strength test, that is, four tennis athletes surpassed the force limits of the ergometer and racing athletes could not firmly grab the rim to produce force. If these athletes could have performed maximal on the isometric strength test, their predicted anaerobic power would be higher, which corresponds with a higher resistance, lower velocity, and probably a higher power output (Veeger, van der Woude, and Rozendal 1991) (Hintzy et al. 2003).

In contrast to the isometric strength test, the sprint test was feasible for all athletes. The regression equation to predict anaerobic power from the mean velocity over 10 s and practiced sport resulted in an *R* ^2^ of 0.84 (Table 2, Figure 5b). Previous research that looked into the association between wheelchair sprint performance and anaerobic power showed weaker associations (*R* ^2^ of 0.59 [van der Scheer et al. 2014] and 0.47 [Soylu et al. 2020]). However, these studies measured the anaerobic power in the lab and the sprint test in the field (van der Scheer et al. 2014) (Soylu et al. 2020). Athletes from the current study performed the sprint and WAnT on the same ergometer, which resulted in more measurement similarities and a higher *R* ^2^. The current *R* ^2^ of 0.84 is higher compared to the earlier equation of T. W. J. Janssen et al. (1993) between the isometric strength and anaerobic power (*R* ^2^ = 0.75) that was used to set the individual's WAnT resistance. Therefore, for future testing in elite wheelchair athletes, it is advised to use the regression equation based on the mean velocity of the 10 s sprint test and practiced sport to predict anaerobic power and to set the individual's WAnT resistance (Table 2, Section 4.3).

### 4.2 Aerobic Graded Exercise Test

Aerobic power was accurately predicted in basketball and tennis athletes and led to valid GXT resistance steps and adequate test durations. In rugby and racing athletes, aerobic power was lower than predicted and led to too high resistance steps and to the lowest test durations (seven or 8 minutes). Rugby athletes showed higher peripheral RPE (9 ± 1) than central RPE (8 ± 1) values, which suggests that they stopped the test mainly because of peripheral reasons (Goosey‐Tolfrey et al. 2014). Although looking at the reasons for ending the test (pain in fingers, cramp), the test duration might be the limiting factor for this group. Future research might consider a shorter steep RAMP test (Rozenberg et al. 2015) or a discontinuous test (Valent et al. 2007) for this group. However, ventilatory thresholds, often used for training guidelines (Wolpern et al. 2015), cannot be derived from these test methods. Secondly, it hampers comparison with athletes from other sport disciplines that are tested with the current test protocol. Besides rugby players, four of the included racing athletes in the current study were pure sprinters who likely have a better anaerobic than aerobic capacity, which also resulted in lower test durations. However, although all athletes (including athletes with a shorter test duration) reached two out of three secondary criteria (RER, HR, and RPE) (Goosey‐tolfrey 2008), their performance was classified as a maximal aerobic exercise. To ensure optimal test durations in future testing, the already existing regression equations can be fine tuned with the newly developed sport‐specific regression equation.

Our multiple regression analysis showed that aerobic power could be predicted best by anaerobic power and practiced sport (*R* ^2^ = 0.78, Figure 5e). The *R* ^2^ solely based on anaerobic power was 0.58 and is lower compared to earlier studies that included a more homogeneous group: T. W. J. Janssen et al. (1993) showed an *R* ^2^ of 0.81 for inactive men with a spinal cord injury whereas, van der Woude et al. (2002) showed an *R* ^2^ of 0.77 for elite male track athletes. The heterogeneous population in the current study is reflected in the significant addition of practiced sport to the regression equation. For better fitting individualized GXT resistance settings, future research in wheelchair athletes is advised to use the regression equation based on anaerobic power and practiced sport as predictor for aerobic power and to set the individual's GXT resistance steps (Table 2, Section 4.3).

### 4.3 Future Recommendations and Practical Implications

There is a large variation in resistance settings for the WAnT and GXT test protocols in international literature (R. J. F. Janssen et al. 2021) and the current standardized, but individualized resistance settings can provide a more uniform way of testing. The current test battery can be implemented by every researcher or practitioner in the same standardized way and no exhaustive test experience or information regarding the athlete is needed to scale the resistance settings to the individual (R. J. F. Janssen et al. 2021). It is advised to perform future testing in elite wheelchair athletes using the updated regression equations to predict (an) aerobic power and to set the individual's resistance settings for the WAnT and GXT (Table 2):

$$P30=–\;1.19+1.02\;*\;\text{sprint}-v_{\text{mean}}\;–\;0.59\;*\;\text{racing}\;–\;0.26\;*\;\text{basketball}\;–\;0.15\;*\;\text{rugby}\;\left(R^{2}=0.84\right)$$

$${\text{PO}}_{\text{peak}}=0.56+0.47\;*\;P30\;–\;0.35\;*\;\text{racing}\;–\;0.01\;*\;\text{basketball}\;–\;0.37\;*\;\text{rugby}\;\left(R^{2}=0.78\right)$$

By having a protocol based on common principles, albeit scaled to the individual's capacity, athletes and coaches will be provided with interathlete and intra‐athlete comparable measurements of their wheelchair‐specific anaerobic and aerobic capacity. These measures can be used to monitor their performance, to give detailed training guidelines (e.g., based on ventilatory thresholds), or to evaluate alterations in wheelchair setup (e.g., wheel/rim size), all in order to ultimately improve their sport performance (Goosey‐Tolfrey et al. 2018).

### 4.4 Strengths and Limitations

The current study included 43 wheelchair athletes from four different sport disciplines, with a large range of impairments, wheelchairs, and personal characteristics, which can be seen as a strength of the current study. The majority of studies examining anaerobic or aerobic capacities focused solely on one sport discipline and chose a test protocol that suits that specific sport discipline, which makes generalization of results to other populations difficult (R. J. F. Janssen et al. 2021). However, the current dataset of 43 wheelchair athletes did not allow to split the group in two, one for regression model development and one for model validation. Therefore, a future study should ideally validate this model before implementing it.

As a limitation of the current study, the test battery was quite exhaustive to perform, which might have led to increasing fatigue during the test battery. Hence, athletes might have been fatigued when starting the last test, the GXT, what potentially could be an additional reason why some athletes ended the test earlier than expected, that is, before 8 minutes. Ideally, the GXT is performed on a separate day, where athletes are not fatigued; however, due to their busy training schedule, it was not possible to perform the testing on two separate days. To minimize the influence of fatigue, 20 min rest after the WAnT was anticipated. Indeed, the WAnT was deemed to be the most strenuous test, and the GXT only started when the athlete indicated to have recovered from this test. A previous study that compared the outcomes of a GXT, with or without performing the WAnT 20 min beforehand, found a significant, but slight decrease in VO_2_peak, from 42.3 (7.6) to 41.4 (7.2) mL·min^−1^·kg^−1^ (Stong et al. 2021). This represents a 2% reduction, which is minimal compared to the day‐to‐day variation in VO_2_peak (13.2%) (Leicht et al. 2013). This suggests that the results in our study are expected to be within normal limits.

Furthermore, as a limitation of this test battery, not every lab has a computerized wheelchair ergometer available. Treadmills can also be used to perform a GXT, but they do not allow isometric strength, sprint, and WAnT testing (de Klerk et al. 2020a). The isometric strength test could be performed on an alternative setup (van der Scheer et al. 2014) and predict aerobic power (*R* ^2^ = 0.74 and Figure 5c). In this way, it remains possible to utilize this standardized and individualized protocol design for the GXT test protocol.

## 5 Perspective

This study evaluated and updated a test battery with regression equations to select standardized and individualized resistance settings for the WAnT and GXT, respectively, to test the anaerobic and aerobic capacity of wheelchair athletes. Researchers and practitioners are advised to use the sprint test as predictor for anaerobic power (i.e., P30 and *R* ^2^ = 0.84) and to set the individual's WAnT resistance. Furthermore, the updated regression equation, based on anaerobic power and practiced sport, is advised to predict aerobic power (i.e., PO_peak_ and *R* ^2^ = 0.78) and to set the individual's GXT resistance steps. The standardized and individualized resistance settings will lead to interathlete and intra‐athlete comparable measures that can be used to, for example, monitor their performance, set detailed training guidelines, or evaluate changes in wheelchair setup.

## Ethics Statement

All procedures performed in studies involving human participants were in accordance with the ethical standards of the institutional and/or national research committee and with the 1964 Helsinki declaration and its later amendments or comparable ethical standards. The local ethics committee of the Centre for Human Movement Sciences, University Medical Centre Groningen, and University of Groningen, the Netherlands approved the study protocol (202000455).

## Consent

Informed consent was obtained from all individual participants included in the study.

## Conflicts of Interest

The authors declare no conflicts of interest.

## Data Availability

The datasets generated during and/or analyzed during the current study are available from the corresponding author on reasonable request.
